# Prevalence of hypertension and diabetes mellitus in Peruvian patients with chronic kidney disease: a systematic review and meta-analysis

**DOI:** 10.1186/s12882-024-03595-x

**Published:** 2024-05-10

**Authors:** Darwin A. León-Figueroa, Edwin Aguirre-Milachay, Joshuan J. Barboza, Mario J. Valladares-Garrido

**Affiliations:** 1 Facultad de Medicina Humana, Universidad de San Martín de Porres, Chiclayo, 15011 Peru; 2 Vicerrectorado de Investigación, Universidad Norbert Wiener, Lima, 15046 Peru; 3 Universidad Continental, Lima, 15046 Peru; 4 Oficina de Epidemiología, Hospital Regional Lambayeque, Chiclayo, 14012 Peru

**Keywords:** Chronic kidney disease, Peru, Hypertension, Diabetes mellitus

## Abstract

**Background:**

Chronic Kidney Disease (CKD) represents a major challenge for public health, with hypertension and diabetes being the main causes of its occurrence. Therefore, this study aims to determine the prevalence of hypertension (HTN) and diabetes mellitus (DM) in Peruvian patients with CKD.

**Methods:**

A systematic search for studies about CKD in Peru was carried out in PubMed, Scopus, Embase, Web of Science, ScienceDirect, Google Scholar, Virtual Health Library (VHL), and Scielo from 2011 to December 2023. The protocol of this research was registered in the international registry of systematic reviews, the Prospective International Registry of Systematic Reviews (PROSPERO), with registration number CRD42023425118. Study selection, quality assessment, and data extraction were performed independently by two authors. Study quality was assessed using the Joanna Briggs Institute Statistical Meta-Analysis Assessment and Review Instrument. A random-effects model with inverse variance weighting was used to estimate the combined prevalence of HTN and DM in Peruvian patients with CKD. To analyze data heterogeneity, the I^2^ statistical test was used. Statistical analysis was performed with R version 4.2.3.

**Results:**

A total of 1425 studies were retrieved, of which 23 were included in the final meta-analysis. A total of 43,321 patients with CKD were evaluated, of whom 52.22% were male and 47.78% were female. The combined prevalence of HTN in Peruvian patients with CKD was 38% (95% CI: 30–46%; 41,131 participants; 21 studies, I^2^ = 99%, *p* = 0), while the combined prevalence of DM in Peruvian patients with CKD was 33% (95% CI: 26–40%; 43,321 participants; 23 studies, I^2^ = 99%, *p* = 0).

**Conclusion:**

Approximately one-third of Peruvian patients with CKD have HTN and DM. These findings highlight the importance of implementing prevention and control measures for these chronic noncommunicable diseases in the Peruvian population, such as promoting healthy lifestyles, encouraging early detection and proper management of hypertension and diabetes, and improving access to medical care and health services.

**Supplementary Information:**

The online version contains supplementary material available at 10.1186/s12882-024-03595-x.

## Introduction

Chronic kidney disease (CKD) is typically defined by a glomerular filtration rate of less than 60 ml/min/1.73 m2 or the presence of other indicators of kidney damage, such as albuminuria [1, 2]. CKD represents a global public health challenge, impacting about 10–14% of the adult population worldwide [3, 4]. Moreover, CKD is closely linked to the increased prevalence of conditions such as diabetes mellitus (DM), hypertension (HTN), obesity, and aging, which continue to be the main causes of morbidity and premature mortality in the population affected by this disease [5, 6].

Diabetes mellitus represents an important risk factor for the development of CKD [7], and several studies have explored its role as an independent risk factor in the incidence of CKD [8, 9]. The prevalence of DM has been steadily increasing worldwide. Currently, about 450 million people suffer from this disease, and this figure is projected to reach 690 million by 2045 [10]. On the other hand, HTN plays a significant role in the onset and worsening of CKD [11]. The prevalence of HTN increases as renal function deteriorates, affecting approximately 60–90% of individuals with CKD [12]. HTN is the most common chronic disease in developed societies and claims about 7.1 million lives worldwide each year [7].

In Peru, the prevalence of CKD in some regions of the country can reach 16.8% [13] to 28.4% in the city of Lima [14]. In addition, it has been observed that among people with CKD, there is a prevalence of DM of 20% and HTN of 55.9% [14]. According to a 2024 press release, the Peruvian Ministry of Health reported that about 10% of adults suffer from CKD, with HTN and DM being the main causes of this condition [15]. In this context, the presence of chronic comorbidities, such as HTN and DM, in patients with CKD not only contributes to the development and progression of kidney disease but also exacerbates the risk of cardiovascular and renal complications, increasing the burden of disease and the costs associated with medical care [16].

In Peru, as in many Latin American countries, the availability of information related to the epidemiology of CKD and its association with other chronic diseases is limited [7, 17, 18]. This field of research is in constant development and is still being explored in depth. Due to its wide diversity in terms of socioeconomic trends, climatic and geographic zones, and social determinants of health (access to health services, economic stability, and education), Peru presents a unique opportunity to assess the burden of CKD [17, 19].

The study aims to fill a significant gap in the understanding of renal health in the Peruvian population, focusing specifically on the prevalence of HTN and DM in patients with CKD. This research not only seeks to quantify the prevalence of these comorbid conditions but may also shed light on the risk factors and possible interactions between CKD, HTN, and DM in this specific context. By providing solid and systematized data, the study could inform more effective health policies aimed at prevention, early detection, and optimal management of these conditions in the Peruvian population, thus improving the care and quality of life of patients with CKD. In addition, it can lay the groundwork for future research and more precise and targeted intervention strategies [20].

## Materials and methods

### Protocol and registration

The present research was conducted following the guidelines of the Preferred Reporting Items for Systematic Reviews and Meta-Analysis (PRISMA) [21] (Table S1), as well as a protocol registered in PROSPERO with the identification number CRD42023425118.

### Eligibility criteria

This review included observational studies, such as cross-sectional studies and prospective and retrospective cohorts, that examined the prevalence of HTN and DM in Peruvian patients over 18 years of age diagnosed with CKD. Studies that did not meet the criteria, such as editorials, letters to the editor, randomized clinical trials, conference abstracts, and narrative or systematic reviews, were excluded.

### Information sources and search strategy

Searches were conducted in various databases, including PubMed, Scopus, Embase, Web of Science, ScienceDirect, Google Scholar, Virtual Health Library (VHL), and Scielo, until December 1, 2023, with no language or development period restrictions. Studies were identified using Medical Subject Headings (MeSH) terms such as “chronic kidney disease” and “Peru”. The search strategy was independently tested by two authors and is detailed in Table S2. In addition, other search methods were used, including a review of literature studies, consultation of article references, and review of publications in Peruvian journals specializing in chronic kidney disease. However, the potential studies identified were within the scope of the search strategy employed.

### Study selection

The search strategy results were stored in the Endnote software. Subsequently, duplicate articles, titles, and abstracts were removed. Next, two investigators independently reviewed the titles and abstracts of the articles to select those that met the inclusion criteria. Then, two additional investigators conducted a thorough review of the full articles to determine if they met the inclusion criteria. Any discrepancies identified were resolved through mutual agreements.

### Outcomes

The main outcome is to determine the prevalence of HTN and DM in Peruvian patients diagnosed with CKD.

### Quality assessment

The JBI-MAStARI tool was employed to assess the quality of the articles included in the meta-analysis. The evaluation encompassed various aspects, such as the study context, outcome and explanatory variables, specific inclusion criteria, measurement standards, topic description, and precise statistical analysis. The quality of the studies was categorized as high (≥ 7 points), moderate (4 to 6 points), or low (< 4 points) based on their scores, and any discrepancies were resolved through researcher discussions (Table S3) [22].

### Data collection process and data items

Three independent researchers were responsible for collecting relevant data from the selected articles and recording it in an Excel spreadsheet. The collected information included various details such as the author, publication year, study design, study location, sample size, and the number of participants with CKD. The prevalence of CKD, study subjects, participants’ age, and gender (both male and female), as well as the presence of HTN and DM were also recorded. Subsequently, to ensure the accuracy and quality of the extracted data, two additional researchers conducted a rigorous review and verification process.

### Data analysis

The data obtained from Excel was utilized for conducting the analysis in R, specifically version 4.2.3. In order to present the research findings, tables and narrative graphs were employed. A random-effects model with inverse variance weighting was used to estimate the combined prevalence of HTN and DM in Peruvian patients with CKD. The Cochrane Q statistic was used to examine the variability among the trials. Additionally, the I2 index was used to quantify this variability. Values of 25%, 50%, and 75%, respectively, were regarded as indicating low, moderate, and high heterogeneity.

A funnel plot was employed to investigate the possibility of publication bias. Egger’s regression test was also used to investigate this matter further. When the resultant p value was less than 0.05, it was believed that there was a possibility of bias in the results.

The pooled prevalence of HTN and DM in Peruvian patients with CKD was shown graphically as a forest plot, with 95% confidence intervals included for enhanced precision in the presentation of the data.

## Results

### Study selection

**Fig. 1:**
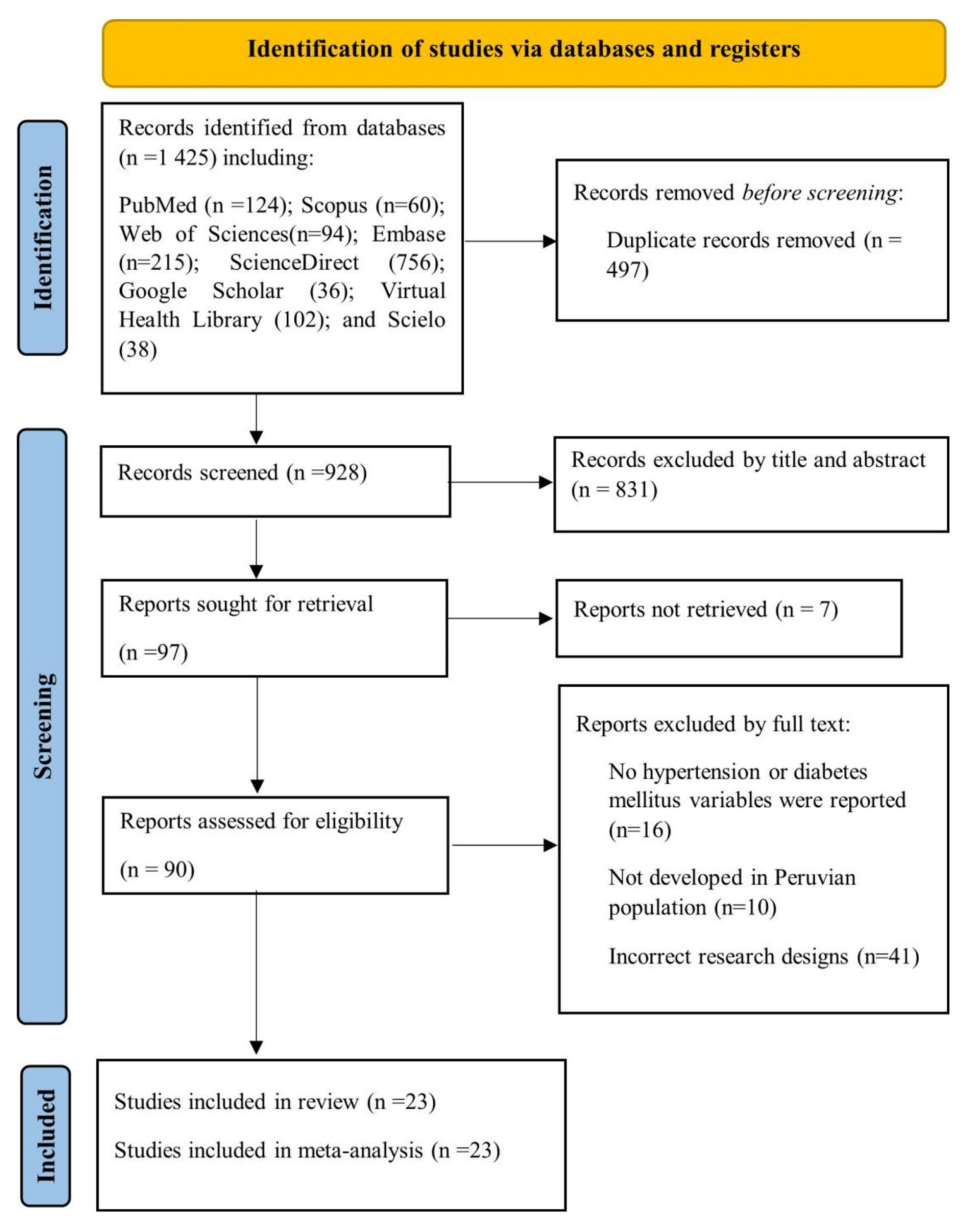
illustrates the process of study selection according to the PRISMA flowchart

A total of 1425 articles were found through searches in eight different databases. The selection process is detailed in the PRISMA flowchart, depicted in Fig. 1. After eliminating duplicate articles (*n* = 497), the investigators analyzed the remaining 928 articles. Subsequently, the titles and abstracts of these articles were evaluated, and 90 were selected for a comprehensive full-text review. Once this process was completed, 23 articles that met the inclusion criteria for the systematic review and meta-analysis were included [14, 17, 23–43].

### Characteristics of the included studie

The analysis was based on a review of 23 observational studies published between 2011 and 2023 that examined the prevalence of HTN and DM in Peruvian patients diagnosed with CKD (Table 1). A total of 43,321 patients with chronic kidney disease were evaluated, of whom 52.22% (22,622) were male and 47.78% (20,699) were female [14, 17, 23–43]. The mean age of the participants was approximately 64 years, and most of the studies were centered in Lima, the capital of Peru (Table 1).

**Table 1:** Characteristics of Peruvian patients with chronic kidney disease in each of the included studies

Authors	Year	Study design	Location	Sample size (*n*)	Chronic kidney disease (*n*)	Prevalence CKD (%)	Study subjects	Age (Years)	Sex		Comorbidities		
									M	F	Hypertension	Diabetes Mellitus	HTN and DM
Meneses Liendo V, et al. [23]	2011	Retrospective cohort	Lima	359	359	100%	General population	> 18	211	148	38	79	NR
Herrera-Añazco P, et al. [24]	2015	Retrospective cohort	Lima	216	216	100%	General population	Mean: 56.9	134	82	20	69	NR
Herrera-Añazco P, et al. [25]	2015	Retrospective cohort	Lima	235	235	100%	General population	Mean: 56.4	149	86	22	78	NR
Francis ER, et al. [17]	2015	Cross sectional	Lima and Tumbes	404	68	16,8%	General population	Mean: 59.6	21	47	29	13	NR
Huamán CL, et al. [26]	2016	Cross sectional	Callao	30	30	100%	General population	Mean: 62.3	17	13	10	11	NR
Bravo-Zúñiga J, et al. [27]	2017	Longitudinal retrospective	Peru	1248	352	28.21%	General population	Mean: 76	199	153	57	280	NR
Gómez de la Torre- del Carpio A, et al. [28]	2017	Retrospective cohort	Lima	557	557	100%	General population	> 18	315	242	295	246	NR
Herrera-Añazco P, et al. [29]	2017	Retrospective secondary analysis	Peru	1211	219	18.08%	General population	Mean: 69	110	109	116	18	66
Pinares-Astete F, et al. [30]	2018	Prospective cohort	Lima	604	604	100%	General population	Mean: 51. 95	342	262	73	121	NR
Huauya-Leuyacc C, et al. [31]	2018	Cross sectional	Peru	148	148	100%	General population	Mean: 55.4	75	73	118	71	NR
Bravo-Zúñiga J, et al. [14]	2019	Cross sectional	Lima	42 746	12 132	28.4%	General population	Mean: 67.7	5644	6488	6785	1701	2423
Herrera-Añazco P, et al. [32]	2019	Cross sectional	Lima Arequipa Ucayali	599	599	100%	General population	Mean: 64	298	301	326	224	NR
Loaiza-Huallpa J, et al. [33]	2019	Retrospective cohort	Cusco	187	187	100%	General population	Range: 43–66	95	92	24	43	NR
Bravo-Zúñiga J, et al. [34]	2020	Prospective cohort	Peru	20 354	20 354	100%	General population	Mean: 72.9	11,060	9294	7877	2795	4335
Pineda-Borja V, et al. [35]	2020	Retrospective cohort	Lima	73	73	100%	General population	Mean: 39	30	43	42	7	NR
Munive-Yachachi Y, et al. [36]	2021	Cross sectional	Peru	155	155	100%	General population	Mean: 63.5	106	49	45	65	19
Valenzuela-Narváez RV, et al. [37]	2021	Cross sectional	Lima	1159	1159	100%	General population	Range: 65–80	534	625	876	752	NR
Umeres-Francia G, et al. [38]	2022	Retrospective cohort	Lima	343	343	100%	General population	Mean: 78.3	216	127	222	103	NR
Guzmán-Ventura W, et al. [39]	2022	Retrospective cohort	Lima	540	540	100%	General population	Range: 49–72	287	253	NR	289	NR
Arellán Bravo LJ, et al. [40]	2022	Retrospective cohort	Junín	128	128	100%	General population	Mean: 59.02	83	45	48	31	NR
Venegas Justiniano JY, et al. [41]	2022	Retrospective cohort	Lima	1650	1650	100%	General population	Mean: 60.51	930	720	NR	711	NR
Herrera-Añazco P, et al. [42]	2023	Retrospective cohort	Lima	3153	3153	100%	General population	Mean: 78.3	1726	1427	572	1294	NR
Huaman-Carhuas L, et al. [43]	2023	Cross section	Callao	60	60	100%	General population	Mean: 56.6	40	20	41	21	NR

NR: Not reported, M/F: Male/Female, CKD: chronic kidney disease, HTN: Hypertension, and DM: Diabetes Mellitus

### Quality of the included studies and publication bias

The quality of the studies was assessed using the JBI Critical Appraisal Tools, specifically designed for cross-sectional research. It was determined that all the studies included in the analysis demonstrated a moderate level of quality, as indicated in Table S3 [14, 17, 23–43]. In the analyses aimed at evaluating HTN in Peruvian patients with CKD, it was observed that when Egger’s test was applied to evaluate publication bias, a value of *p* = 0.5339 (t = -0.63, df = 19) was obtained. This result suggests that the null hypothesis of symmetry is accepted, indicating that there is no evidence of publication bias in the studies examined (Figure S1). In the analyses aimed at evaluating DM in Peruvian patients with CKD, it was evident that when Egger’s test was used to evaluate publication bias, a value of *p* = 0.0063 (t = 3.04, df = 21) was obtained. This result indicates an asymmetry in the data, which could explain the considerable disparities in the reported prevalence values. However, it should be noted that we were unable to conclusively demonstrate the presence of publication bias (Figure S1).

### Joint prevalence of HTN and DM in Peruvian patients with CKD

**Fig. 2:**
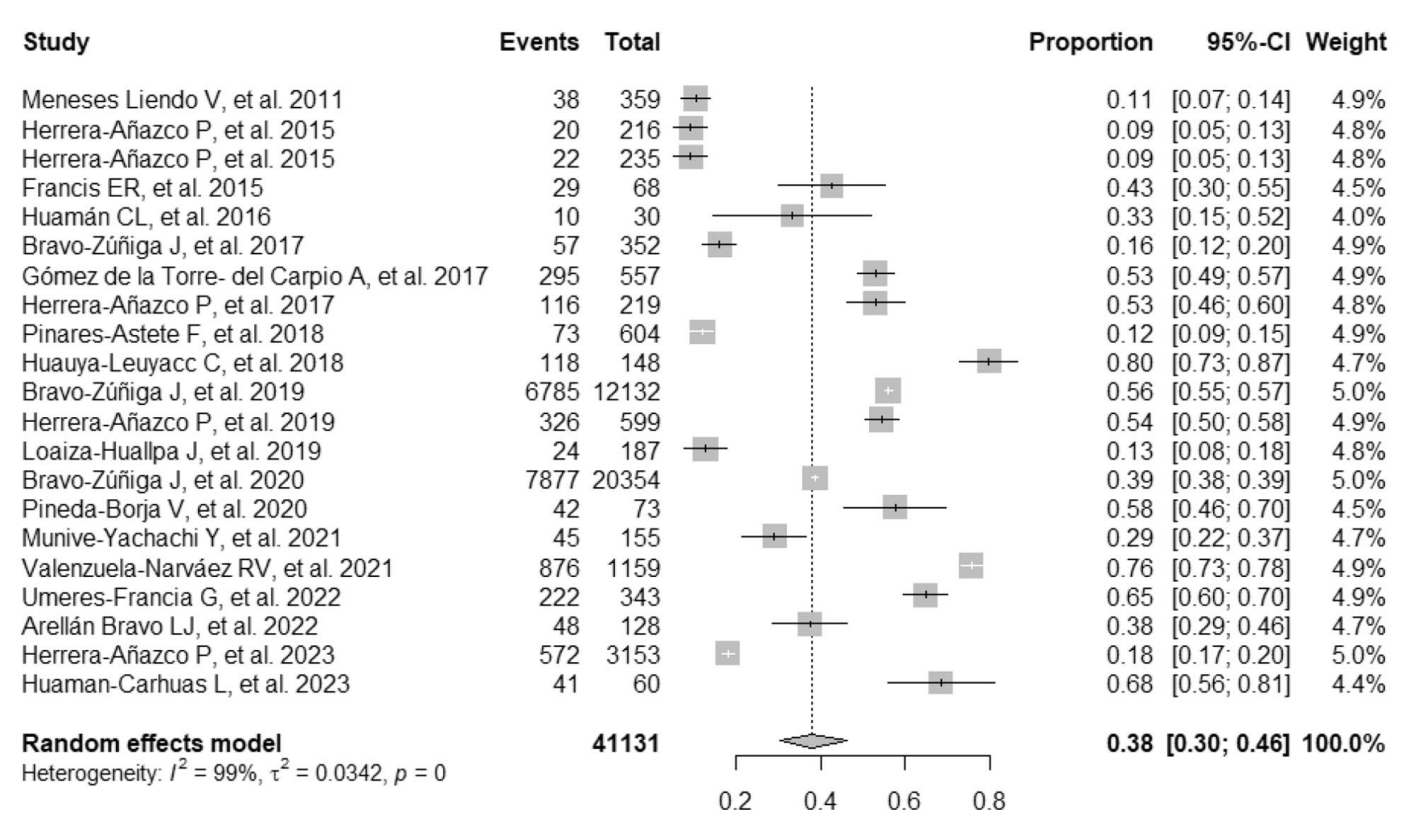
Forest plot illustrating the joint prevalence of Hypertension in Peruvian patients with chronic kidney disease

**Fig. 3:**
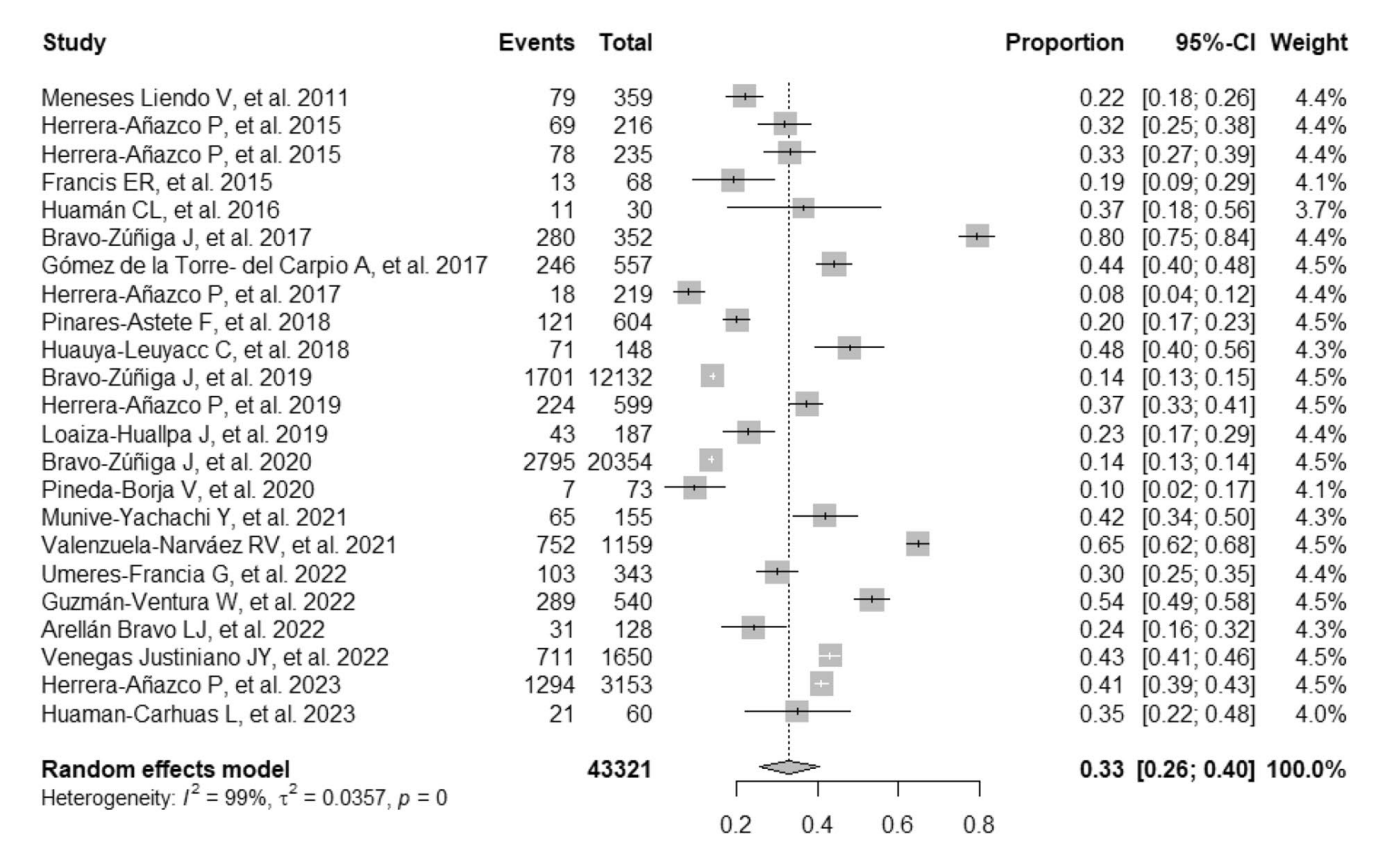
Forest plot illustrating the joint prevalence of diabetes mellitus in Peruvian patients with chronic kidney disease

The combined prevalence of HTN in Peruvian patients with CKD was 38% (95% CI: 30–46%; 41,131 participants; 21 studies). The I^2^ test indicated significant heterogeneity among the included studies (I^2^ = 99%, *p* = 0) (Fig. 2) [14, 17, 23–38, 40, 42, 43]. The combined prevalence of DM in Peruvian patients with CKD was 33% (95% CI: 26–40%; 43,321 participants; 23 studies). The I^2^ test indicated significant heterogeneity among the included studies (I^2^ = 99%, *p* = 0) (Fig. 3) [14, 17, 23–43]. The combined prevalence of HTN and DM in Peruvian patients with CKD was 21% (95% CI: 19–23%; 32,860 participants; 4 studies). The I^2^ test indicated significant heterogeneity among the included studies (I^2^ = 89%, *p* = 0) (Figure S2) [14, 29, 34, 36].

## Discussion

Chronic kidney disease constitutes a challenge of great relevance for public health at a global level, with an ever-increasing incidence and prevalence. In addition to its considerable medical and economic burden, CKD implies a notable increase in morbidity and mortality rates among the affected population. CKD is a non-communicable disease generally caused by diabetes and hypertension [44]. Therefore, the purpose of this systematic review and meta-analysis was to determine the prevalence of HTN and DM in Peruvian patients with CKD. The most relevant results showed that the combined prevalence of HTN in this population reached 38%, while the combined prevalence of DM was 33%.

The Centers for Disease Control and Prevention’s CKD Surveillance System reported that about 14% of adults in the United States have CKD stages 1 to 4 [45]. Kovesdy CP reported that CKD affects more than 10% of the general population worldwide and is more prevalent in older people, women, racial minorities, and people who experience DM and HTN [3].

In a meta-analysis by Hill NR, et al. reported an overall prevalence of 5-stage CKD of 13.4%, and stages 3–5 was 10.6% [46]. In addition, the prevalence of individual stages of CKD was 3.5% (stage 1), 3.9% (stage 2), 7.6% (stage 3), 0.4% (stage 4), and 0.1% (stage 5) [47]. According to the National Health and Nutrition Examination Survey (NHANES), the prevalence of CKD among adults aged 70 years and older was lower in 2017–March 2020 (42.6%) than in 2001–2004 (52.1%) [48]. Hill NR, et al. reported a linearly higher prevalence for CKD stages 1–5 associated with advancing age, ranging from 13.7% in the 30–40 year age group to 27.9% in patients > 70–80 years [46].

A study by Sundström J. et al. in 2.4 million patients from 11 countries reported a CKD prevalence of 10% [49]. In Asia, the prevalence of stage 3–5 CKD in low- and middle-income countries was 11.2% [50]. Another study found that 14% of the general population and high-risk groups in South Asia had CKD [51]. In Peru, Bravo-Zúñiga J, et al. reported a prevalence of CKD of 28.4% in patients evaluated in a health network in the city of Lima [14]. Another study by Herrera-Añazco P. et al. reported a CKD prevalence of 18% [29].

The combined prevalence of HTN in Peruvian patients with CKD was 38%. In the United States, the prevalence of CKD stages 1–4 among hypertensive adults was 26.34% in 2017–2020, compared with a prevalence of 7.8% among nonhypertensive individuals [52]. Hill NR et al., in their meta-analysis, reported an association between HTN and CKD prevalence [46]. In Tanzania, Stanifer JW et al. reported that among adults with CKD, 19.3% had HTN [53]. Another study found that the prevalence of CKD was 27% in adults with HTN [51]. In Peru, Bravo-Zúñiga J. et al. evaluated a total of 20,354 participants with CKD; 38.7% had HTN [34]. These results are based on the fact that HTN is a medical condition distinguished by elevated blood pressure, which is a significant risk factor in the development and progression of CKD. This condition can cause damage to the blood vessels in the kidneys, compromising their ability to efficiently filter waste and excess fluids from the body [54].

The combined prevalence of DM in Peruvian patients with CKD was 33%. In the United States, according to NHANES (2017–2020), the prevalence of CKD stages 1–4 in diabetic adults was 38.67% [55]; in addition, the prevalence of CKD stages 3–4 (NHANES 2001–March 2020) was 10% among adults with prediabetes or undiagnosed DM and 18% among adults with diagnosed DM [56]. A study by Fernandez-Fernandez L. et al. in Spain reported a CKD prevalence of 25.3% in patients with DM [57]. Gatwood J. et al. reported that CKD was evident in 31.6% of veterans before being diagnosed with DM [58]. A meta-analysis by Hill NR et al. reported an association between DM and the prevalence of CKD [46]. In Tanzania, Stanifer JW et al. reported that among adults with CKD, 7% had DM and 14.0% had DM and HTN [53]. Another study found that the prevalence of CKD was 31% in adults with DM [51]. Sundström J. et al. reported that DM was present in 38% of patients with CKD [49]. In Peru, Bravo-Zúñiga J, et al. evaluated a total of 20,354 participants with CKD; 13.74% had DM [34]. Diabetes is positioned as one of the leading causes of CKD, triggering a number of nephropathic complications. This disorder exerts a significant influence, as elevated blood glucose levels cause progressive damage to the small blood vessels that supply blood to the kidneys. This detrimental interference compromises kidney function, creating an environment conducive to the development and aggravation of kidney disease [59].

The present study has some limitations. First, the included studies may be subject to biases and confounding factors that cannot be individually quantified. Second, it was not possible to perform a subgroup analysis by gender because the studies provided only baseline demographic data for the CKD population. Third, the prevalence of HTN and DM according to CKD stages could not be determined. Finally, the sample sizes of the included studies varied considerably, ranging from 20,354 to only 30 participants. However, among the strengths of this study, it is noteworthy that this research represents the first systematic review and meta-analysis focused on the evaluation of HTN and DM in Peruvian patients with CKD. To guarantee the quality of the included studies, the “JBI-MAStARI” tool was used as the evaluation method. In addition, a specific search strategy was designed for each database, and article selection and data extraction were carried out independently by two or more investigators. It should be noted that the studies included in this review shared the same definitions of CKD, and the recommendations established by the PRISMA guidelines were rigorously followed.

## Conclusions

Approximately one-third of Peruvian patients with CKD have HTN and DM. These findings highlight the importance of implementing prevention and control measures for these chronic noncommunicable diseases in the Peruvian population, such as promoting healthy lifestyles, encouraging early detection and proper management of hypertension and diabetes, and improving access to medical care and health services.

### Electronic supplementary material

Below is the link to the electronic supplementary material.

Supplementary Material 1

## Data Availability

No datasets were generated or analysed during the current study.
